# Covid-19, the polarization of substance use, and mental health^⋆^

**DOI:** 10.1016/j.jped.2024.04.003

**Published:** 2024-04-26

**Authors:** Jürgen Rehm

**Affiliations:** a Institute for Mental Health Policy Research, Centre for Addiction and Mental Health, Toronto, Ontario, Canada; b Campbell Family Mental Health Research Institute, Centre for Addiction and Mental Health, Toronto, Ontario, Canada; c PAHO/WHO Collaborating Centre at CAMH, Toronto, Canada & WHO European Region Collaborating Centre at the Public Health Institute of Catalonia, Barcelona, Catalonia, Spain; d University of Toronto, Dalla Lana School of Public Health, Toronto, Ontario, Canada; e University of Toronto, Faculty of Medicine, Department of Psychiatry, Toronto, Ontario, Canada; f University of Toronto, Institute of Medical Science, Faculty of Medicine, Toronto, Ontario, Canada; g Center for Interdisciplinary Addiction Research (ZIS), Department of Psychiatry and Psychotherapy, University Medical Center Hamburg-Eppendorf (UKE), Hamburg, Germany; h Program on Substance Abuse & WHO European Region Collaboration Centre, Public Health Agency of Catalonia, Barcelona, Catalonia, Spain

In a cohort of Brazilian adolescents, assessed for substance use and mental health before and during the COVID-19 pandemic, Dr. Garcia-Cerde and colleagues recently found a decrease in past-year and past-month substance use frequency, despite some adolescents initiating, maintaining, or even increasing the frequency of their substance use.^1^ Moreover, these authors found that changes in increased alcohol use in particular were associated with mental health problems. This is yet another example of the polarization of substance use in times of crisis, which had been predicted at the beginning of the pandemic,^2^ based on earlier systematic reviews of the literature published during times of economic crises or natural catastrophes (e.g.,^3^^,^^4^).

What does polarization mean and how can it be explained? First of all, economic crises or natural catastrophes lead to a reduction in availability and/or affordability of substances. In the case of COVID-19, lockdowns, restaurant and bar closures, disruptions of the supply chains for illicit drugs, and similar measures were used to curb the spread of the pandemic. All of these processes are known to reduce the level of consumption of substances. In the case of legal substances, the restriction of availability or affordability of substances is even known as “best buys” for policy by the World Health Organization.^5^ Restrictions of availability also happened for illegal drugs during the pandemic where even though the overall drug supply and trafficking proved to be resilient to COVID-19-related measures, there was a decrease in supply and demand.^6^ However, the increase in mental stress for many people during the pandemic also led to an increase in substance use in some groups, especially the most vulnerable groups, including those that had been more heavily using alcohol and illegal drugs prior to the crisis caused by the pandemic.^2^ Finally, these mechanisms were predicted to cause more severe substance-attributable health consequences, in particular amongst the heaviest users.

Literature reviews corroborated the above-described polarization effects. For instance, with respect to alcohol consumption, the following reviews indicated that, during the Covid-19 pandemic, those, who had consumed heavily before—including but not limited to people with alcohol use disorders—increased their consumption, and a large part of the population decreased their consumption due to availability restrictions.^7^, ^8^, ^9^, ^10^, ^11^ Also, it became evident that despite lower levels of use, substance-attributable mortality increased (e.g., for alcohol in Europe:^12^; for illegal drugs:^6^). The substance-attributable mortality may have increased as a direct consequence of COVID-19-related measures, including measures that have led to reduced access to medical services or equipment for illegal drug use, an increase in risky behavior, such as injecting alone or reduced access to support networks and safer spaces in which to use drugs (for example, supervised consumption and treatment service settings). Moreover, people who use legal or illegal drugs may be at higher risk with regard to catching COVID-19.

So the work of Garcia-Cerde and colleagues^1^ fits perfectly into the emerging literature on the polarization of substance use during Covid. However, two features distinguish their findings: first, their sample was restricted to adolescents, where in general you would expect some gradient of increasing levels of substance use with increasing age. And, secondly, they explicitly included assessments of mental health indicators, using a validated instrument, the Strengths and Difficulties Questionnaire (SDQ). The SDQ is a screening measure used to assess emotional or behavioral difficulties and prosocial skills for 4- to 17-year-olds. It comprises a total scale of five subscales: emotional symptoms, conduct problems, inattention-hyperactivity symptoms, problems with peers, and prosocial behavior.

The main result was that those who started their alcohol consumption were more likely to present behavioral problems (SDQ total), as well as symptoms of conduct disorders and inattentive-hyperactivity problems, which were considered at a clinical case level. So, changes in starting alcohol drinking at the third follow-up were associated with behavioral problems, whereas stopping had no association with these levels and maintaining consumption was only linked to conduct disorders. However, these regressions were not controlled by prior SDQ scores. It could be that people scoring high on the scale for conduct disorders initiated and increased their alcohol use, and continued to show high scores on this scale, just to give one potential explanation.

Moreover, the study comprised people who were either in the experimental or the control group of a drug prevention program, and it is unclear how this status impacted the results. The linear effect of group status was adjusted for in the described results; however, there may have been interactions between group status and other variables.

In sum, while the authors observe an association between alcohol use changes and indicators of mental health by the third follow-up, the potential causality remains unclear. It will be important to:

a) predict which people increase their alcohol consumption during situations where in general there is a decrease in consumption; as well as
b) further examine the causality and the complex relationships between different patterns of substance use and mental health indicators.

These two questions are not only important for adolescents, but concern the behavior of other groups, and the whole population during crisis situations such as Covid-19 as well. However, despite these suggestions for future research, the work of Garcia-Cerde and colleagues^1^ gives important insights into substance use behaviors during a crisis situation such as COVID-19.

## Conflicts of interest

The author declares no conflicts of interest.
